# Extended Implicit Bias: When the Metaphysics and Ethics of Implicit Bias Collide

**DOI:** 10.1007/s10670-021-00511-9

**Published:** 2022-01-12

**Authors:** Uwe Peters

**Affiliations:** 1 Center for Science and Thought, University of Bonn, Bonn, Germany; 2 Leverhulme Centre for the Future of Intelligence, University of Cambridge, Cambridge, UK; 3 Department of Psychology, King’s College London, London, UK

## Abstract

It has recently been argued that to tackle social injustice, implicit biases and unjust social structures should be targeted equally because they sustain and ontologically overlap with each other. Here I develop this thought further by relating it to the hypothesis of extended cognition. I argue that if we accept common conditions for extended cognition then people’s implicit biases are often partly realized by and so extended into unjust social structures. This supports the view that we should counteract psychological and social contributors to injustice equally. But it also has a significant downside. If unjust social structures are part of people’s minds then dismantling these structures becomes more difficult than it currently is, as this will then require us to overcome widely accepted ethical and legal barriers protecting people’s bodily and personal integrity. Thus, while there are good grounds to believe that people’s biases and unjust social structures ontologically overlap, there are also strong ethical reasons to reject this view. Metaphysical and ethical intuitions about implicit bias hence collide in an important way.

## Introduction

Whether it is in the recurring killings of unarmed Black Americans (like George Floyd) by White policemen,^1^ the sexism in Hollywood (see #MeToo movement) or in EU politics (see the EU’s recent ‘sofa gate’),^2^ the xenophobic attacks on migrants and Asians (in the wake of COVID-19),^3^ ableism, or ageism, social injustice is too common. How can we best combat it?

Philosophers working on this question often focus on two factors contributing to social injustice. The first is *implicit bias*, which refers broadly to largely unconscious and/or automatic cognitions linking the members of a social group with one or more negative (or positive) characteristics (implicit stereotype), or a negative evaluation (implicit prejudice) (Fitzgerald et al., 2019). Implicit bias is commonly taken to be one key factor contributing to social injustice (Beeghly & Madva, 2020). The second one is *social structure*, referring broadly to the social institutions (family, government, education, etc.), social roles (gender, profession, etc.), status (middle-class, student, etc.), and social norms that maintain order in a group by guiding, limiting, and organizing behavior (House, 1981). Social structure can be unjust, restricting some individuals or groups unfairly, and promoting discriminatory perceptions, attitudes, and behavior (Madva, 2020).

To what extent should we focus on individuals’ implicit biases, unjust social structures, or both to eradicate social injustice? In the literature on this issue, three groups of philosophers can be distinguished: individualists, structuralists, and integrationists. *Individualists* hold that people’s individual minds with their implicit and explicit biases are the root cause of and should be our primary focus in tackling social injustice (Blum, 2002; Garcia, 1996). In contrast, *structuralists* hold that social structures are its main cause and should be our main focus of attention (Anderson, 2010; Haslanger, 2015). *Integrationists* are ecumenical, maintaining that minds and social structures are intimately intertwined (or integrated with each other) and equally involved in causing social injustice, meaning that both should equally be targeted with interventions (Ayala-Lopez & Beeghly, 2020; Davidson & Kelly, 2020; Leboeuf, 2020; Madva, 2020; Soon, 2020).

All three, individualists, structuralists, and integrationists, agree that appeals to *both* mental states and social structures are necessary for an adequate explanation of persistent social ills. The difference between the camps is in explanatory priority and opinion on whether biased minds or unjust social structures are more deeply engrained or robust.

The focus here will be on integrationists. To support their view, several integrationists have recently proposed that implicit biases and social structures ontologically overlap in that they are partly constitutive of each other. For example, Leboeuf (2020) holds that “implicit biases should not be conceived of as [entirely] ‘inside the head’ of individuals, but rather as [partly] *social*” (p. 41).^4^ Similarly, Ayala-Lopez and Beeghly (2020) suggest that to the “extent that […] social biases make social relations what they are, they partially constitute these relations”, and since individuals absorb social structure, we should think of people’s “biases […] as a way in which the social structure manifests in” them (p. 216). Relatedly, Davidson and Kelly (2020) hold that some social “structures” are “fluidly interwoven with” and “internalized” by an individual, “existing (in part) as psychological states […] in her brain” (p. 200). Soon (2020), too, argues that people’s biased minds are coupled to certain unjust social structures such that the two “really constitute one system” (p. 1872).

One might be an integrationist (i.e., hold that minds and social structures should enjoy equal explanatory priority and be equal targets of interventions) without committing to the metaphysical view that biased minds and social structures ontologically overlap. However, the focus here will be on philosophers who do advocate this view, and, more generally, on theorists who aim to soften the ontological boundaries between the two entities. These researchers are henceforth the sole referents of the term ‘integrationists’.

The integrationists’ assumption that implicit bias and social structure ontologically overlap can be understood as a version of Clark and Chalmers’ (1998) *hypothesis of extended cognition*, which posits (HEC): Some human cognitive states or processes extend into, i.e., are partly realized by, objects or processes outside the body (for details, see Rowlands et al., 2020). Indeed, some integrationists briefly refer to HEC in passing (Davidson & Kelly, 2020, p. 209; Soon, 2020, p. 1873). However, no integrationist has so far explicitly committed to any version of HEC. And while HEC has been applied to various cognitions (Rowland et al., 2020), it remains unclear whether the most common conditions for extended cognition proposed by advocates of HEC would also in some cases support the postulation of an *extended implicit bias*. Moreover, the potential implications of adopting such a conceptualization of implicit bias remain largely unexplored.

In the following, I want to change this by arguing for three main points:

1. Individuals and their implicit biases often interact with unjust social structures in ways that meet common conditions for extended cognition, specifically, extended implicit bias. That is, the literature on HEC provides reasons to believe that people’s implicit biases are often partly realized by unjust social structures.
2. If implicit biases are often partly realized by unjust social structures, this helps support the integrationists’ point that minds and social structures are equally implicated in social injustice. But it also makes it more difficult to reduce social injustice. Because if unjust social structures are partial realizers of people’s minds, then they are in these cases literally external components of people's minds. And so to be able to dismantle these structures, we will need to overcome widely accepted ethical and legal barriers protecting individuals’ bodily and personal integrity. Indeed, racists and their ilk could use widely accepted ethical and legal frameworks to hold that currently perfectly legitimate policy changes to tackle unjust social structures are in fact instances of personal assault on them.
3. This unattractive upshot creates a hitherto unnoticed conflict between metaphysical and ethical considerations for integrationists and advocates of HEC. On the one hand, recent integrationist arguments and common conditions for extended cognition provide grounds to believe that people’s biases extend into certain social structures. Yet, on the other hand, there are ethical reasons to reject this notion, as it makes eradicating unjust social structures more difficult than it currently is.

The support for (1)–(3) rests partly on the assumption of conditions for extended cognition. Since there are various objections to HEC (Adams & Aizawa, 2008; Rupert, 2004), I will offer arguments for a particular set of conditions for extended cognition that provides a response to some of these objections.

However, making a case for HEC isn’t the project here. Correspondingly, I won’t commit to HEC but will settle with a conditional conclusion: *If* we endorse the main considerations that integrationists offer for the ontological overlap of minds and social structures and accept common conditions for extended cognition then the assumption of the existence of extended implicit bias is supported. While I will also suggest that we can't easily dismiss the antecedent, even if we remain doubtful about HEC, the argument below will still help advance the theorizing on implicit bias, social justice, and HEC. This is because it illustrates that the recent proposal to view implicit bias as partly located in social structures raises significant and difficult ethical challenges that have so far gone unnoticed by integrationists (and advocates of HEC).

Notice that in argumentatively supporting an external extension of a pernicious cognition such as implicit bias that we have good ethical reasons to reduce, I don’t mean to suggest that implicit bias is somehow desirable or worthy of enlarging. In fact, the goal here is exactly the opposite. It is to help refine the research on how we can best eradicate implicit bias and unjust social structures by making explicit the potential problems of conceptualizing implicit bias as an (in some cases) extended cognition.

In Sect. 2, I introduce the main considerations that integrationists have proposed for a partial ontological fusion of biased minds and social structure. In Sects. 3 and 4, I develop the integrationists’ thought further by relating it to work on HEC and arguing for claim (1). In Sects. 5–7, I make the case for claims (2)–(3).

## Minds and Social Structure as One System

Integrationists commonly begin motivating their view by emphasizing that any community contains social norms and expectations signaling group boundaries and indicating how to properly identify and interact with occupants of different social roles. Some of these social structures might be materialized in, for instance, cultural artefacts. Consider pictures of a mother being nurturing to her child, an image capturing a stereotype familiar from novels or movies. Such artefacts structure social interactions by specifying, restricting, and influencing how mothers act (Ayala-Lopez & Beeghly, 2020). Other elements of social structure include explicit formal norms, and familiar but frequently tacit guidelines, unwritten rules, or verbally conveyed customs organizing a community’s social interactions (Davidson & Kelly, 2020).

Social structures, in general, can be more or less fair to those occupying certain social roles (by limiting, e.g., women’s leadership prospects). But either way, integrationists note, people internalize them because this allows them to smoothly enter into social interactions within their community and become intrinsically motivated to conform to the group (ibid). The problem is, integrationists add, that when the internalized structures are unfair to certain groups or social roles, this internalization results in unjust social structures eventually coming to “manifest in” people’s biases (Ayala-Lopez & Beeghly, 2020, p. 216). That is, social structures have a “double life”: They are both public resources binding individuals to groups and internal states guiding cognition (Davidson & Kelly, 2020, p. 202).

Importantly, integrationists continue, cognition and social structures aren’t independent of each other but in “cycling loops of mutual influence” (ibid, p. 198): Biases aren’t only produced and reinforced by social structures encountered in social interactions, novels, movies, and so on, but themselves also produce and reinforce these structures, in turn, by influencing people’s behavior, expectations, interactions, etc. Given this interdependence, there are no “sharp boundaries […] between individuals and structures” (ibid, p. 205). Rather, to some extent, “biases make social relations what they are” and so “partially constitute these relations” just as social structures, in turn, partially ‘make’ and manifest in the biases (Ayala-Lopez & Beeghly, 2020, p. 216). While integrationists generally agree that the ontological boundaries between minds and social structures should thus be “softened” (Davidson & Kelly, 2020, p. 205), not all explicitly argue that both ontologically overlap.

Some do, however (e.g., Leboeuf, 2020; Soon, 2020). Soon (2020) holds that implicit biases result from learning associations formed across repeated stimuli pairings, eventually becoming memory schemas that can emerge as irreducible group products from social interactions. For example, after repeatedly hearing politicians claim ‘Muslims hate Western culture’, people may create a label ‘Muslim’ that they then link with ‘hating Western culture’. Suppose that in social interactions, one person has so far only connected Muslims with hating Western culture while another person only associates ‘Muslims’ and ‘terrorists’. In communication, the two people might then conclude ‘Muslims are terrorists *because* they hate Western culture’. Soon argues that since this new association doesn’t reduce to each individual’s association or their conjunction, its emergence can’t be attributed to either individual alone but requires postulating a system that comprises both and their interaction.

Moreover, the resulting new association can subsequently become a social structure, a *social schema*, existing in language, images, or behavioral patterns, Soon continues. Social arrangements, interactions, and individuals’ thoughts can then either strengthen or reduce the signals that such schemas send, thereby producing new structural elements, consolidating, or counteracting existing ones. After all, people don’t just reflect internalized schemas back into the world, but create and change them such that the schemas would be different if not for the minds and behavior of individuals. Echoing other integrationists, Soon holds that there is thus an ongoing “dynamic feedback loop” between minds and structure (2020, p. 1873). People’s interactions create social schemas, which become internalized into people’s heads, which again influence the schemas, and so on.

Drawing on dynamical systems theory (Palermos, 2014), Soon adds that since the schemas and their effects on people’s minds are partly determined by people’s own effects on the schemas, the schemas and their effects aren’t entirely exogenous (i.e., originating from outside) but partly endogenous to people’s minds. And since the effects of structure on minds and the effects of minds on structure thus partly overlap, the “division between [the] two apparently distinct entities [i.e., mind and social schema] dissolves”: “implicit bias both causes and partly constitutes a form of social schema”, i.e., social structure, Soon concludes (2020, p. 1866).

While her view implies that social schemas are part of people’s biased minds, Soon doesn’t yet consider the notion of an extended implicit bias. But the just mentioned dynamical systems theoretical considerations are in fact frequently used to argue for cases of extended cognition, or so I will show next.^5^

## Arguments and Conditions for Extended Cognition

In their argument for HEC, Clark and Chalmers (1998) ask us to imagine two characters: Otto, an Alzheimer’s patient who relies on a notebook to remember information, and Inga, a neurotypical individual. One day, Otto and Inga decide to go to a museum. Inga retrieves the museum’s address from her memory and goes there. Otto first consults his notebook, finds the museum’s address, and goes there too.

Clark and Chalmers suggest that the notebook plays for Otto the same role that memory plays for Inga in that it guides action and is accessed when required. There is thus, they continue, a basis for holding that the notebook physically realizes Otto’s belief about the museum’s address, because what “makes some information count as a belief is the role it plays, and there is no reason why the relevant role can be played only from inside the body” (1998, p. 14). More generally, Clark and Chalmers endorse a

(1) “parity principle”: “If, as we confront some task, a part of the world functions as a process which, were it done in the head, we would have no hesitation in recognizing as part of the cognitive process, then that part of the world is […] part of the cognitive process” (ibid, p. 8).

Anticipating the worry that (1) might sanction untenably far-reaching extensions of cognition into, for example, libraries (this worry is also known as the “cognitive bloat” objection to HEC; Rowland et al., 2020), Clark and Chalmers proposed several further conditions for an artefact *A* to qualify as a component of *S*’s mind: *A* also needs to be:

(2) reliably available and commonly used for a given task, and

(3) the information provided by *A* has to be automatically accepted and

(4) easily retrievable by *S* (Clark, 2010, p. 46).

However, Adams and Aizawa (2008) responded that even with (1)–(4) in place, and if *A* and *S* are thus tightly causally connected, this doesn’t mean that *A* is then also *constitutive* of *S*’s cognitive system; inferring otherwise is a “coupling-constitution fallacy”. To address this objection, Clark and others introduced additional motivations for HEC by citing the kind of considerations from dynamical systems theory already touched on above. This theory holds that two systems produce one extended system if there are ongoing bidirectional feedback loops between the contributing parts (Clark, 2008, pp. 80, 131; Chemero, 2009). To support this, Palermos (2014) introduces two points:

- Continuous bidirectional interactions “give rise to new systemic properties that belong only to the overall system and to none of the contributing subsystems alone. Therefore, to account for these new systemic properties, one has to postulate the overall extended or distributed system” (ibid, p. 33).
- In “cases of ongoing feedback loops between the coupled systems, there is dense non-linear causal interdependence that disallows us to decompose systems in terms of distinct inputs and outputs from the one to the other” since the “effects of each component to the other are not entirely endogenous to the affecting component, and vice versa”: The “effects of the environment on the agent are *partly determined* by [i.e., not originating entirely from outside] the agent’s own ongoing activity at that time, and vice versa. It is, therefore, impossible to decompose the ongoing causal effects in terms of distinct inputs and outputs from the one system to the other” (ibid, p. 34).

Points (a)–(b) are often used to motivate the following additional condition for extended cognition:

(5) For *A* to be part of *S*’s cognitive system, *A* and *S* need to be in an ongoing reciprocal interaction (feedback loops) with each other (Clark, 2008; Palermos, 2014).

Some advocates of (5) emphasize that for cognitive extensions the “relevant reciprocal interactions need only be continuous during the operation of the relevant coupled cognitive system and the unfolding of any processes related to it” (Palermos & Tollefsen, 2018, p. 121). Moreover, condition (5) and points (a) and (b) are thought to offer a response to the ‘cognitive bloat’ objection to HEC: people aren’t in any obvious sense in continuous reciprocal interactions with, say, libraries. These points are also taken to explain why postulating extended systems needn’t involve a ‘coupling-constitution fallacy’: If *S* and *A* exert effects on each other that can’t adequately be viewed as entirely exogenous inputs to (or fully endogenous outputs from) either *S* or *A*, and produce properties that can’t be ascribed to either *S* or *A* alone, then this supports the constitution claim.

However, I will remain agnostic on whether condition (5) and points (a) and (b) in fact allow advocates of HEC to avoid the mentioned objections. I also won’t commit to conditions (1)–(5).

What matters here is only that some or all of conditions (1)–(5) are often endorsed in the literature on HEC. And this is indeed the case. Especially condition (5) is currently very popular and advocated by many philosophers as a sufficient criterion for cognitive extensions, including—importantly—extensions into *abstract aspects* of social environments, for instance, group dynamics, social practices, or legal structures (e.g., Alfano & Skorburg, 2017; Carter et al., 2017; De Jaegher, 2013; Gallagher, 2013; Palermos, 2016; Palermos & Tollefsen, 2018). That is, these theorists hold that not only concrete physical objects but also abstract social structures can be realizers of cognition if condition (5) is met (all the just cited papers endorse this move).^6^ For instance, Alfano and Skorburg (2017) hold that “when an agent is functionally integrated through ongoing feedback loops with her social environment, the environment doesn’t just causally influence her but becomes [a constitutive] part of her character [and so her self], for good or ill” (p. 468). The notion of extended implicit bias, however, hasn’t appeared in this context yet.

## Revisiting Implicit Bias and Social Structures

It remains unclear whether social structures are ever used for any cognitive task such that, in these cases, the interaction(s) between people’s implicit biases and social structures meets conditions (1)–(5) for extended implicit bias. Since exploring the matter might advance our understanding of the nature of the two entities, I will now turn to it.

To begin with, people do frequently rely on social structures (social roles, etc.) materialized in texts, images, or cultural practices for identifying ways of thinking, feeling, and acting that allow them to interact smoothly with members of their community (Davidson & Kelly, 2020). To ensure smooth interactions, people need to stay in sync with the social structures in their community, which presupposes an ongoing monitoring of them (Pickett & Gardner, 2005). In fact, already the point that social structures guide and influence implicit biases on an ongoing basis suggests that people often turn to their social environment(s) (texts, images, behavioral patterns, etc.) to retrieve, and factor in, social structure when engaging in social cognition. Does the use that people make of social structures in these cases ever meet condition (1) for extended implicit bias?

Consider a situation in which one has encountered unfair gender roles in the media, movies, conversations, or people’s behavior, and inadvertently internalized them. It seems clear that when they are subsequently in social cognition unconsciously retrieved from memory and affect judgment- and decision-making, we would view this as implicitly biased cognition. If so, then by the parity principle, it should also count as such when the information about these unfair gender roles, or any other aspect of social structure, is not retrieved from inside the head but ‘read off’ from one’s environment (e.g., before internalization) as part of one’s ongoing monitoring of social structures and their manifestations. That is, there is reason to believe that the interactions between people’s biases and social structures (in individuals’ continuous monitoring of the latter as part of their social cognition) meet condition (1) for extended implicit bias.

Turning to conditions (2)–(4), various unjust social structures (e.g., gender roles, social schemas, etc.) are (unfortunately) reliably available in people’s social environments, manifesting in people’s language use in their community (incl. online groups), texts, movies, and so on (Ayala-Lopez & Beeghly, 2020). There might be no specific physical object (like Otto’s notebook) through which unjust social structures are available to people. But this also needn’t be the case. For recall that many advocates of HEC have already argued that *abstract social structures* (incl. social norms and behavioral patterns) too can be realizers of extended cognition (e.g., Alfano & Skorburg, 2017; Carter et al., 2017; De Jaegher, 2013; Gallagher, 2013; Palermos, 2016; Palermos & Tollefsen, 2018), and the argument here is conditional on these points.

Moreover, the here relevant unjust social structures clearly are frequently readily used in social cognition, because we generally (unconsciously) conform to our community’s social norms, roles, etc., as indicated in our generally smooth social interactions (and IAT scores), suggesting that these structures are reliably tracked and acted upon. Additionally, many people generally do trust and (e.g., during their development) automatically endorse some (if not most) elements of social structure. In fact, as some integrationists argue, our cognitive system might have evolved to absorb such structures (e.g., social norms), as this is adaptive (Davidson & Kelly, 2020). Finally, unjust social structures are also generally easily accessible in our communities, media images, the news (e.g., when Muslims are overrepresented as terrorists, Dixon & Williams, 2015), or inequalities at work. In short, there is reason to believe that at least in some cases unjust social structures meet conditions (2)–(4) for counting as realizers of extended implicit bias.

Notice that even if implicit bias sometimes counts as extended into social structures, this doesn’t mean that the bias itself is then also fully accessible to the individual. After all, some internal components of the bias might remain introspectively undetectable even if other parts of it are extended and publicly available, i.e., the term ‘extended *implicit* bias’ isn’t an oxymoron.

Turning finally to condition (5), integrationists already emphasize that the interactions between minds and social structures do often involve “feedback loops of mutual influence” (Davidson & Kelly, 2020, p. 198; Soon, 2020, p. 1872). The idea is that implicit biases currently have the persistent profile and specific contents they do partly because they are in reciprocal feedback loops with unjust social structures. Notice that this is compatible with granting that implicit bias and unjust social structures might also to some extent exist independently.

Relatedly, recall that according to (5), for extended cognition, the reciprocal interactions between *A* and *S* need only be continuous during the operation of the relevant coupled system, not all the time (Palermos & Tollefsen, 2018, p. 121). For instance, when a Facebook user sees various ‘likes’ of information capturing negative social schemas in her favourite Facebook group, she might (unconsciously) endorse and add content to them in her subsequent exchanges (posts, ‘likes’, shares, etc.) with others on the website. This might happen during a single 2 min Facebook check, or gradually over time (e.g., weeks). During these interactions, a coupled system would be instantiated in which the Facebook user and the social structures at issue continuously reciprocally interact (on Facebook). Indeed, Soon (2020) relies precisely on such considerations to argue that biases and social schemas “constitute one system” (p. 1872).

It is important, however, to distinguish between the *producers* and the *targets* of pernicious social schemas here: the producers are people (or structures) that impose the schemas on other individuals, i.e., the targets. Crucially, the feedback loops between problematic social schemas (e.g., negative stereotypes) and their *targets* are perhaps often largely asymmetric and unidirectional, as targets may frequently have little power over and influence on the schemas. These loops, one might hold, are hence different from those at issue in condition (5).

But consider instead interactions between social schemas and powerful, privileged individuals who are not the schemas’ targets and who (due to their status) have significantly more control over the schemas and (intentionally or unintentionally) produce them. For instance, social media influencers, celebrities, or politicians might be part of online groups where ideologically similar people listen to them, and readily pick up/transform their social associations by affirming and adding to each other’s views. In these cases, minds (especially, those of the powerful individuals) and social schemas do influence each other in a more balanced and bidirectional manner, and their interaction may thus frequently meet condition (5).

This point can be extended from social schemas to social structures more generally because, as noted, we don’t just follow institutions, social roles, and so on but engage with, interpret, and construct them. When social structures are, through contextualized interpretation, sufficiently stretched, they can and do get changed, suggesting that people are in a relationship of bidirectional influence with them. Again, the bidirectional influence will be more (or less) limited for some individuals, in some environments, with respect to some elements of social structure, than for others. For instance, some social roles or norms at people’s workplace might be much more restrictive than others, permitting only limited influence by those subject to them. People’s influence on social structure might also in some situations be much more temporally stretched than in others (e.g., instantaneous structural changes might be rare or minute). The bidirectional influencing of minds and structure, where the effects of one on the other are partly endogenous to the affected part, is thus a matter of degree. Still, it is real (e.g., even seemingly rigid legal structures can be changed by lawyers, judges, etc. setting precedents; Gallagher, 2013). There are thus more or less balanced and bidirectional continuous feedback loops between social structures, in general, including unjust ones, and the minds operating within (and on) them. This provides reasons to believe that, especially among influential, privileged, or powerful individuals, the interactions between the two entities frequently meet condition (5).

In short, we have grounds to hold that these interactions often satisfy all five conditions for extended implicit bias and so, if we endorse conditions (1)–(5), then such cognitive extensions exist. In fact, the monitoring of environments for social structure and the engagement with at least some of its elements (e.g., at work, or on social media) is a pervasive process in people’s social cognition to avoid being socially excluded and to signal belonging (Pickett & Gardner, 2005). Many people will thus perhaps frequently interact with social structures in the ways outlined above, making it plausible to assume that extended implicit bias is common.

These points support the integrationists’ case that biased minds and unjust social structures are equally important to tackle in order to eradicate social injustice. This is because the notion of extended implicit bias helps to see that structural interventions (e.g., policies against social inequalities, social schemas in movies, etc.) can be *psychological* interventions on extended minds.

## An Argument Against Extended Implicit Bias

The preceding section supported integrationist claims to the effect that “implicit biases should not be conceived of as [entirely] ‘inside the head’ of individuals, but rather as [partly] social” (Leboeuf, 2020), i.e., biases “partially constitute” unjust social structure (Ayala-Lopez & Beeghly, 2020, p. 216), and so both partly “constitute one system” (Soon, 2020, p. 1872). I shall now mention a problem with the metaphysics of implicit bias underlying these claims. That is, I will introduce an argument to reject the notion of extended implicit bias. It takes the following form:

(P1) According to widely accepted ethical and legal frameworks, intentionally dismantling a part of a person that is responsible for (i.e., partly realizes) that person’s (mental or physical) faculties is (generally) personal assault when it happens without the person’s consent.

(P2) If a person *S*’s implicit bias extends into certain unjust social structures, then these structures are parts of *S* responsible for her (mental) faculties.

(C1) If *S*’s implicit bias extends into certain unjust social structures then, according to widely accepted ethical and legal frameworks, intentionally dismantling these structures is (generally) personal assault when it happens without *S*’s consent. (*Modus ponens* from (P1)+(P2).)

(P3) Intentionally dismantling these unjust social structures via policy changes is *not* (generally) personal assault according to any ethical or legal framework.

(C2) *S*’s implicit bias does *not* extend into unjust social structures. (*Modus tollens* from (C1)+(P3).)

Skeptics of HEC might take (P1)–(C2) to be just another *reductio* of HEC. However, the argument has broader implications because it equally applies to claims by integrationists that implicit biases are partly constituted by social structure. Moreover, as noted, especially condition (5) for extended cognition is currently popular among many philosophers. Since that condition implies extended implicit bias, if (P1)–(C2) is on track, these philosophers too will have grounds to reconsider their stance.

### Supporting premise (1)

Consider (P1). Carter and Palermos (2016) offer good reasons for endorsing this premise. They note that personal assault is commonly understood as a type of harm that implicates the use of force to another individual’s body without her consent. Current ethical and legal frameworks support this. Ethically speaking, the freedom from such a violation of bodily integrity is widely taken to be central to one’s right to self-ownership and personal autonomy (Cohen, 1995; Quinn, 1993). As Quinn (1993) puts it: “A person is constituted by his body and mind. They are parts or aspects of him. For that very reason, it is fitting that he have primary say over what may be done to them—not because such an arrangement best promotes overall human welfare, but because any arrangement that denied him a say would be a grave indignity” (p. 170).

Similarly, legally speaking, assault has for a long time been viewed as a more serious category of offence than, say, damage to an individual’s property (Blitz, 2010). For instance, US police officers are allowed to search suspects and the area immediately surrounding them without a warrant. But they can’t search an individual’s physical interior without warrant (Carter & Palermos, 2016).

What counts as ‘interior’? Common ethical and legal frameworks don’t view only the body with its organic shell as potential subject of inadmissible interferences but tend to favor a functionalist understanding of a person and her faculties (ibid). For instance, Blitz (2010), a law scholar, notes that we “value our mental capacities, not simply the particular machinery or resources that make them possible. If so, it makes sense to protect not only the internal biological resources crucial for their exercise, but other resources as well” (p. 27).

Relatedly, the US Supreme Court ruled in 2014 that in some cases, even cell phones count as sufficiently integrated into a person (the “proverbial visitor from Mars might conclude they were an important feature of human anatomy”)^7^ such that searches of data on the phones require a special warrant (Riley vs. California, 2014). That is, since cell phones are often highly integrated into an individual’s cognition and behavior, in these cases, these artefacts are treated (by the US Supreme Court) as on a par with parts of one’s own body and so under special protection from non-consensual inspection and interference. Building on these (and other) points, Carter and Palermos (2016) argue that when external resources that qualify as part of one’s extended cognitive system and so of one’s self^8^ are “intentionally compromised” without one’s consent, this “qualifies as a case of personal assault” (p. 549).

This claim needs to be moderated, however, because ‘intentionally compromising’ or dismantling the realizer of a person’s faculties without that person’s consent doesn’t always count as assault. The debate on compulsory COVID-19 vaccination to protect a country’s population offers an example. Douglas et al. (2020) note that interferences with the right to bodily and personal integrity “can be justified if they are in accordance with national law, pursue a legitimate aim and are proportionate in relation to this aim. In the case of vaccinations or treatments intended to stem the spread of a pandemic disease, a legitimate aim is present” (p. 2). Intentionally compromising a part of a person responsible for that person’s functioning is thus generally, but clearly not always, treated as personal assault even when it happens without the person’s consent.

It should be noted that there are also arguments against “self-ownership”, i.e., the idea that it is the moral/natural right of an individual to have bodily integrity and be the exclusive controller of their own body (e.g., Lippert-Rasmussen, 2008, 2018).^9^ But these arguments can be set aside here. This is because the present point is just that in current ethical and legal frameworks, the right to bodily integrity is widely accepted, and a rejection of this right is thus not unproblematic.

Indeed, especially when it comes to inferences with a person by the government, in Western democracies, strong legal constraints do ensure protection of bodily integrity. For instance, in the literature on the European convention of human rights, one finds statements to the effect that the “inner world of the person lies outside the jurisdiction of the state” (Harris et al., 2009, p. 428). Similarly, returning to bodily integrity and a government’s potential compulsory treatment or vaccination for COVID-19, in “English law, the competent individual’s right to refuse any medical intervention that interferes with her body is well established and enjoys strong protection” (Douglas et al., 2020, p. 1). While “mental health law provides some exceptions to this right”, for “most individuals who possess decision-making capacity, the right persists even when the individual’s reasons for refusing an intervention are bizarre, irrational or non-existent, when undergoing the intervention would clearly be in her best interests, and indeed when refusing the intervention would certainly lead to her death” (ibid). Against this backdrop, it is plausible to assume that widely accepted ethical and legal frameworks treat the intentional dismantling of a part of a person that is responsible for that person’s faculties (whether it is her own body or mind) generally as an instance of personal assault when it happens without the person’s consent.

### Supporting premise (2)

Consider now (P2), i.e., that if *S*’s implicit bias extends into certain unjust social structures then these structures are parts of *S* responsible for her (mental) faculties. (P2) is definitional. If *S*’s implicit bias extends into unjust social structures, then, in line with the arguments from Sects. 3–4, this just means that these structures partly realize her bias and so become a part of *S* responsible for her (mental) faculties. After all, *S*’s implicit biases are parts of her body and self that are partly responsible for her social cognition, which is a mental faculty.

It might be suggested that at best only the mental faculties (e.g., the automatic associative, and perceptual processing of social norms and patterns) that *generate* implicit bias as their content or output are extended into unjust social structures, not the bias itself. However, this overlooks the integrationist argument that implicit biases are partly literally internalized unjust social structures (i.e., bias and structure “constitute one system”; Soon, 2020, p. 1872). It also overlooks the arguments for extended cognition from Sects. 3–4—e.g., the point that, since we would view the information that social structures carry as part of the cognitive system when it is retrieved from internal memory, we should also view it as part of the cognitive system when it is instead retrieved from the social structures (provided the other conditions from Sect. 3 are met too). One might reject these arguments. But since (P2) is conditional on them, this wouldn’t undermine inference (P1)–(C1).

Still, it might be objected that argument (P1)–(C1) fails to do justice to the point that what makes non-consensual interference with gadgets plausibly qualify as cases of assault is that they are *personal* artefacts, i.e., objects we own. By contrast, social structures are more like intellectual commons such as, for instance, Wikipedia. And if, say, I rely on Wikipedia regularly to form beliefs and this satisfies conditions for extended beliefs, others can surely still legitimately edit Wikipedia without my consent even if this results in changing my extended beliefs, because I have no ‘ownership’ claim to Wikipedia. Or so the objection concludes.

However, whether others might still legitimately edit Wikipedia without my consent when I’m using it to form beliefs in ways satisfying conditions (1)–(5) is at issue. And notice that the current ethical and legal frameworks mentioned above presuppose a notion of a person. They don’t settle whether something counts as part of a person and don’t require, for instance, that an artefact be *owned* by a person to be part of that person. That is, the normative frameworks that settle whether something is personal assault build on an existing concept of a person. And so *if* Wikipedia is literally part of my mind and person then they will indeed protect me from the envisaged interferences (for a development of this idea, see Peters, 2021).

We might hold that ‘ownership’ is a condition on something to count as part of someone’s mind and person. But we would then also reject the notion of extended implicit bias, as individuals don’t own social structures, and so the point above would agree with the conclusion of argument (P1)–(C2). Alternatively, if we grant this notion then the mentioned normative frameworks could be used to motivate a *revision* of our conception of ownership of, for instance, intellectual commons. Indeed, drawing on Locke’s ‘labor theory’ of property, some advocates of HEC have argued that if conditions of cognitive extensions are met, then individuals’ close engagement even with abstract social structures can *create* property rights, as their work enters into the object, turning the object gradually into their property (Gallagher, 2013, p. 9).

I needn’t commit to any of these views here because, as noted, (C1) is a conditional: *if S*’s implicit biases extend into certain unjust social structures then by widely accepted ethical and legal frameworks, intentionally dismantling these structures is (generally) personal assault when it happens without *S*’s consent. I shall thus take the first part of overall argument (P1)–(C2) to be supported.

### Supporting premise (3)

Turning finally to (P3), there are many ways of intentionally dismantling unjust social structures, ranging from “less to more transformational and impactful—and, accordingly, from less to more controversial (Madva, 2020, p. 247). For instance, a company’s policy to review job applicants’ CVs anonymously would dismantle some unjust social structure if it was previously common practice in the company to process CVs with names visible, resulting in unfair decision-making. Similarly, to tackle social schemas, a university might require intergroup cooperation among students to reduce in-group vs. out-group stereotypes (e.g., White, Black, Muslim, Christian students collaborating on projects) (ibid). Other ways of counteracting unjust social structures include affirmative actions, diversity quotas for companies, or movies,^10^ tearing down memorials that reinforce racism, or, perhaps most radically, starting a socialist revolution distributing a nation’s resources more equally.

Towards the radical end of the spectrum, there might be egalitarian interventions that involve personal assault (think of the Pol Pot regime; Kiernan, 2008). But they aren’t relevant here. The focus is on the other mentioned kinds of (more or less) collectively supported structural changes, i.e., requirements for anonymous review, intergroup cooperation, affirmative action, deconstructing memorials, diversity quotas, etc. Clearly, these kinds of intentionally dismantling unjust social structures aren’t personal assault by any ethical or legal framework. (P3) is therefore well supported. If so, then this provides a basis for denying the antecedent of (C1), that is, *S*’s implicit biases do not extend into unjust social structures.

## Implications

It is worth emphasizing that, in Western democracies, when activists try to promote or implement the here relevant kinds of changes, perhaps most people will agree with them, as unjust social structures are typically viewed as pernicious. Similarly, when politicians put in place or promote policies to dismantle unjust social structures (e.g., institutions, social roles, norms), this doesn’t generally happen without people’s consent. The public can vote for or against politicians in elections and so give or withhold consent to policies.

Unfortunately, not all people reject social injustice. Racists, bigots and their ilk are unlikely to consent to many changes geared towards tackling unjust social structures, as they may benefit from these structures. We might discount their objections as misguided. But the problem is that if their implicit biases literally extend into unjust social structures of the kind mentioned then these people can in their effort to prevent social change appeal to widely accepted ethical and legal frameworks that protect individuals’ bodily and personal integrity. Concretely, they could try to insist that activists’ proposed policy changes such as anonymous CV review, inter-group cooperation, affirmative action, deconstructing racist memorials, diversity quotas for the media (to tackle social schemas), and so on involve a dismantling of parts of their own (extended, biased) minds and selves without their consent.

If racists, more generally, people disinclined towards social-justice interventions lacked control over the relevant social structures, we could perhaps reject their claim by holding that the mutual mind-structure feedback loops involved would likely be too imbalanced to meet, for instance, condition (5) for cognitive extension. However, the recent rise of populist, often xenophobic political parties worldwide^11^ suggests that in perhaps many social settings, biased people dismissive of social justice interventions may also be in positions of power and significant control over unjust social structures. When it comes to these people (or think of, e.g., Nazi Germans and their shocking schemas of Jews in the 1940s), the view that condition (5) is met becomes particularly challenging to reject.

Granted, bodily and personal integrity is only *one* basic right. It might be overruled by or be much weaker than others. For instance, if unjust social structures persist, this, too, will infringe on people’s rights, namely their right to social justice. This harm, one might argue, is greater than that related to the violation of someone’s bodily and personal integrity by non-consensual alterations of parts of their extended mind. But this may not always obviously be the case. As noted, the right to bodily and personal integrity currently already provides strong barriers to other policies that serve the social good, for instance, compulsory COVID-19 vaccination (Douglas et al., 2020). Similarly, if unjust social structures are parts of (biased) minds, i.e., integral components of persons, then opponents of changes to those structures might hold that these changes, too, infringe on their bodily and personal integrity.

Of course, if the preceding argument is on track then the unjust social structures relevant here aren’t just part of a particular racist’s mind but also part of the minds of all other (more reasonable) people interacting with these structures in ways meeting conditions (1)–(5). We might thus hold that racists should have no more right to influence what happens to these structures than everyone else affected by (and interacting with) them.

The problem remains, however, that to eradicate unjust social structures via collectively enforced policy changes without coming in conflict with current ethical and legal frameworks, we would still need to seek people’s individual consent (vs. their collective consent, which we could obtain via elections, etc.) to interferences with their bodily and personal integrity. Again, many egalitarians will perhaps view implicit biases as unwanted parts of their mind and would readily consent to the interferences at issue.^12^ To intentionally dismantle unjust structures without consent may then only involve a potential personal assault from the point of view of racists, bigots, etc. But the trouble is that it isn’t clear whether, once we ask the public for this kind of consent, most people will indeed transfer decisions over parts of their extended inner world to elected officials or activists in the way they are currently happy to transfer decisions on public goods (social institutions, etc.) to them. That is, if unjust social structures are literally part of the minds and selves of all individuals who are in continuous feedback loops with them (and so should have a say on what happens to these structures), this creates more uncertainty and new ethical and legal hurdles for us to overcome to be able to tackle social injustice with currently perfectly legitimate means.

Put differently, at the moment, people don’t think of the social structures governing their social interactions as constitutive parts of their minds and selves but as merely external resources. Construed in that way, unjust social structures are more easily modifiable with policy making and activism than on the extended implicit bias view. Because if implicit biases sometimes extend in the way outlined above, then eradicating social injustice via activism, electing officials, or policy making means at least in some cases literally tweaking the *realizers* of people’s mind. This is an action akin to brain surgery and so an intervention that many people are usually strongly opposed to and ethically and legally protected from (for related points in the context of neuroethics and online manipulation, see Levy, 2007; Lippert-Rasmussen, 2018; Peters, 2021). That is, the intervention would be significantly different from changing people’s mind by, for instance, persuading them with arguments, ‘nudging’ them (Thaler & Sunstein, 2008), or even ‘brainwashing’ people via persistent exposure to pre-selected, biased information. For all these other methods are still only *indirect*, happening from ‘outside’ people’s minds. They aren’t invasive in the physical sense (though they might involve infringements of privacy; ibid). Alterations of the realizers of people’s minds, however, are. If social justice interventions on social structures amount to such alterations, this is thus likely to elicit stronger resistance to them than to any of the more familiar ways of changing people’s minds.

In contrast, if implicit bias does *not* extend into social structures, these concerns won’t arise. Activists won’t face additional ethical or legal hurdles because no one (in a Western democracy) is currently ethically or legally protected from, say, policy changes aimed at reducing social injustice (even if one disagrees with these changes and the latter reduce people’s biases, thus indirectly altering their minds). Integrationists have therefore grounds to reject the notion of extended implicit bias.

## The Metaphysics and Ethics of Implicit Bias Collide

On the face of it, rejecting HEC might not be too bad. The idea of extended cognition is hardly uncontroversial. However, this move is problematic for several recent integrationists. This is because they are committed to the view that people’s biases and certain social structures are partly constitutive of each other (Soon, 2020; Leboeuf, 2020; Ayala-Lopez & Beeghly, 2020), or at least not strictly ontologically distinct (Davidson & Kelly, 2020). Moreover, since the interactions between the two meets five common conditions for extended cognition, many advocates of HEC are now committed to the notion of extended implicit bias too. Indeed, just focusing on condition (5), as noted, many researchers now endorse the idea that “when an agent is functionally integrated through ongoing feedback loops with her social environment, the environment doesn’t just causally influence her but becomes part of her character, for good or ill” (Alfano & Skorburg, 2017, p. 468; Carter et al., 2017; Gallagher, 2013; Palermos, 2014, 2016; Palermos & Tollefsen, 2018). This view—in conjunction with the integrationists’ point that bias and social structure mutually influence each other in precisely such loops—implies extended implicit bias. So, there are many philosophers who can’t easily reject this notion.

They might respond that the feedback loops between biased minds and unjust social structures are “imbalanced”, “unidirectional”, or “exploitative” and so don’t count as realizing extended cognition proper (Skorburg, 2017). But this response only works well if we focus on the targets of such structures, i.e., people negatively affected by and largely powerless against them. If we focus on the (negatively) potentially largely unaffected producers of them—which unfortunately are often those in power (e.g., think of White, male, Westerners dominating company leadership positions)—then there are many social situations in which we are likely to find more balanced feedback loops between mind and structure. In fact, the more powerful and privileged individuals are the higher the likelihood that they are in a significantly bidirectional, reciprocal connection with the social structure they are interacting with. Hence, precisely those people whom we would want to prevent the most from making the claim that unjust social structures are literally part of their minds and selves are also the ones for whom the imbalance consideration is less convincing.

This point matters because it helps address an important objection to the above argument. This objection is that even if dismantling unjust social structures violates the racist’s right to bodily integrity, *not* dismantling these structures also violates the *anti*-racist’s right to bodily integrity. After all, their social cognition, too, arguably extends into social structures, and the persistence of unjust aspects of these structures may undermine their cognitive functioning. Thus, the objection continues, the right to bodily integrity may, in fact, weigh in favor of changing unjust structures, as long as the racists do not have some property that grounds the claim that such changes are uniquely or particularly an assault on them, providing them with a special entitlement over unjust structures (compared to the non-racists).

The preceding consideration on balance vs. imbalance in mind-structure interactions suggests an answer to this objection. For there arguably are at least some social environments in which racists and their ilk do have a (regrettably) special property: in some social environments (e.g., as a result of historical injustice), they tend to be the ones significantly more in power and control of the relevant unjust social structures (e.g., via their control over media) than those negatively affected by them (for some relevant examples, see Peiser, 2020; Bheeroo et al., 2021).^13^ Because of this difference in power and control, the racists’ mind-structure interactions are then more balanced, and so the metaphysical claim that their biased minds literally extend into these structures is more supported (given conditions (1)–(5)) than the corresponding claim by anti-racists. By extension, in these contexts, racists’ claims to being assaulted by changes to unjust social structures may become more tenable than the deeper competing claims of those who desire interventions.

Given this ethically highly unattractive upshot, integrationists and advocates of HEC might wish to retreat to the view that social structures are at best only scaffolding (i.e., causally coupled to) and not literally extending implicit biases. But the problem is that it isn’t clear that once we grant the ongoing feedback loops between minds and social structures that integrationists highlight, we can easily dismiss the reality of extended implicit bias. And these loops are indeed hard to deny because individuals’ interactions and minds *do* (some more than others) produce social structures (schemas, etc.), which become internalized into people’s heads, which then again influence these structures, and so on. As noted, in these loops, the influence of social structure on biased minds is at least to some extent grounded in and originating from biased minds’ influence on these structures (Davidson & Kelly, 2020). It thus isn’t easy to conceive of the effects of the affecting part as merely causal input into the affected part. For to the extent that the effects of the affecting part (e.g., social structure) come partly from within the affected part itself (e.g., a biased mind), they can’t be viewed as coming purely from without the affected part. And to the extent that they are coming from *within* the affected part, they can't plausibly be construed as merely *causal* effects on but emerge as (to some extent) constitutive of that part, meaning that (in the cases at hand) biased minds and social structure ontologically overlap.

I’m not committing to this dynamical systems theoretical point here. I reiterate it only to highlight that rejecting this kind of support for the ontological-overlap assumption is challenging. When we try to sharply demarcate inputs from outputs within mind and structure interactions, there will be limits to the plausibility of doing so, if we want to do justice to the dynamic interdependence of minds and structure. However, if we grant that there is *some* ontological overlap between them, it seems we are left with the admission of an extended implicit bias and the related significant ethical problem outlined above. There is thus a clash between metaphysical and ethical concerns, especially for recent integrationists and advocates of HEC. For on the one hand, there are seemingly plausible metaphysical considerations to endorse the view that people’s implicit biases extend into social structures, making the latter part of people’s minds. Yet, on the other hand, there are good ethical grounds to reject the view that people’s biases are partly realized by social structures, as this seems to enable, for instance, racists to invoke plausible normative frameworks to undermine social justice efforts in hitherto impossible ways.

Integrationists and advocates of HEC might be able to resolve this conflict between the metaphysics and ethics of implicit bias. It might well be that there is a way in which we can do justice to both the dynamic interdependence of minds and social structure, and the idea that they are merely causally connected even in cases where we focus on powerful individuals with significant control over the relevant elements of social structure. It might also be that the “ontological entanglement” between mind and structure highlighted here can’t be individuated as a specific type of entity (Marmodoro, 2011). Or upon scrutiny, current ethical and legal conceptions could turn out to allow for the kind of interventions on extended minds introduced above. Whether this is so remains to be seen. The present paper achieves its goal if it brings the problem into view and prompts critical reflection on it.

## Conclusion

People’s implicit biases and society’s unjust structures play key roles in maintaining social injustice. Several integrationists argue that both should have equal priority in accounting for and tackling injustice because they are in ongoing feedback loops influencing each other such that they come to overlap ontologically. I developed this proposal by arguing that if we assume five popular conditions for extended cognition, then people’s implicit biases are often partly realized by and so extended into certain social structures. I noted that this view helps to argue that in eradicating social injustice, we should attend to biased minds and unjust social structures equally. But it comes at a price, because if this view is correct, then we will also need to overcome additional ethical and legal concerns so as to be able to alter unjust social structures as easily as we currently can. There is thus an ethical reason to reject the notion of extended implicit bias. Yet, as indicated, this notion can’t easily be dismissed because *prima facie* reasonable metaphysical considerations support it. Hence, while I shall not commit to the notion of extended implicit bias nor HEC here, I conclude that for integrationists sympathetic to a partial ontological fusion of minds and social structures and for advocates of HEC, there is a situation in which metaphysical considerations about implicit bias are in significant tension with ethical considerations.

## Footnotes

1 https://www.bbc.com/news/topics/cr07gpl05emt/us-police-killings.

2 https://www.cbsnews.com/news/sofagate-charles-michel-ursula-von-der-leyen/.

3 https://time.com/5797836/coronavirus-racism-stereotypes-attacks/.

4 If not otherwise indicated, all italics in the quotes of this paper are original. I have added the qualifiers 'entirely' and 'partly' here based on my interpretation of Leboeuf's view. Leboeuf might be read as committing to the even stronger view that implicit biases are not in the head at all.

5 Some parts of Sect. 3 were adopted from Peters (2021).

6 In fact, in their literature review, Rowlands et al. (2020) note that there has recently been a “*social turn*” in work on HEC towards arguments that “social structures and institutions might also, in part, be constitutive of mental processes”.

7 See https://www.law.cornell.edu/supremecourt/text/13-132.

8 One might challenge the view that the extension of a cognitive system also implies an extended self and person (Olson, 2011). However, Carter and Palermos and many other advocates of HEC either assume, or explicitly argue for exactly this implication, and respond to objections against the move from HEC to extended selves (Anderson, 2008; Heersmink, 2017). I won’t repeat the arguments here but will take the move to be sufficiently motivated.

9 Interestingly, Lippert-Rasmussen (2018) uses the assumption of HEC (i.e., the idea that external objects can literally be part of an individual’s self and person) to argue against self-ownership. While he doesn’t consider implicit bias but focuses on extended minds in general, on his view, the argument outlined above would perhaps not yield a *reductio* of extended implicit bias but a *reductio* of the view that we possess rights to bodily integrity. This is one possible response to the argument. But, as noted, it would be in tension with the widely accepted notion that people do have a right to bodily integrity. For the purpose of my argument, all that matters is that this notion is indeed commonly endorsed (as it is). I’m grateful to an anonymous reviewer for pointing me to Lippert-Rasmussen (2018).

10 https://www.nytimes.com/2020/06/12/movies/oscars-diversity-rule.html.

11 The BBC offers a helpful country-by-country guide to the rise of right-wing nationalism: https://www.bbc.com/news/world-europe-36130006.

12 This doesn’t undermine (C1). Because even if anti-racists would not view the interventions at issue as personal assault on them, it would still be the case that according to widely accepted ethical and legal frameworks, it would count as such.

13 For specific here relevant US population data, see also https://worldpopulationreview.com/us-city-rankings/most-racist-cities-in-america.
